# Metabolomics analysis of *Lactobacillus plantarum* ATCC 14917 adhesion activity under initial acid and alkali stress

**DOI:** 10.1371/journal.pone.0196231

**Published:** 2018-05-24

**Authors:** Wenwen Wang, Jiayi He, Daodong Pan, Zhen Wu, Yuxing Guo, Xiaoqun Zeng, Liwei Lian

**Affiliations:** 1 Key Laboratory of Animal Protein Deep Processing Technology of Zhejiang, Marine Science School, Ningbo University, Ningbo, Zhejiang, P. R. China; 2 Department of Food Science and Nutrition, Ginling College, Nanjing Normal University, Nanjing, P. R. China; 3 Ningbo Dairy Group, Ningbo, Zhejiang, China; Agricultural University of Athens, GREECE

## Abstract

The adhesion ability of *Lactobacillus plantarum* affects retention time in the human gastro-intestinal tract, as well as influencing the interaction with their host. In this study, the relationship between the adhesion activity of, and metabolic changes in, *L*. *plantarum* ATCC 14917 under initial acid and alkali stress was evaluated by analyzing auto-aggregation, protein adhesion and cell adhesion *in vitro*. Based on scanning electron microscope (SEM) and transmission electron microscope (TEM) analysis, the morphology of the bacteria became thickset and the thickness of their cell walls decreased under initial alkali stress. The fold changes of auto-aggregation, adhere to mucin and HT-29 cell lines of *L*. *plantarum* ATCC 14917 in the acid group were increased by 1.141, 1.125 and 1.156, respectively. But decreased significantly in the alkali group (fold changes with 0.842, 0.728 and 0.667). Adhesion—related protein increased in the acid group but declined in the alkali group at the mRNA expression level according to real time polymerase chain reaction (RT-PCR) analysis. The changes in the metabolite profiles of *L*. *plantarum* ATCC 14917 were characterized using Ultra-Performance Liquid Chromatography-Electrospray ionization-Quadrupole-Time of Flight-mass spectrometry (UPLS-ESI-Q-TOF-MS). In the alkali group, the content of a lot of substances involved in the energy and amino acid metabolism decreased, but the content of some substances involved in the energy metabolism was slightly increased in the acid group. These findings demonstrate that energy metabolism is positively correlated with the adhesion ability of *L*. *plantarum* ATCC 14917. The amino-acids metabolism, especially the amino acids related to pH-homeostasis mechanisms (lysine, aspartic acid, arginine, proline and glutamic acid), showed an obvious effect on the adhesion ability of *L*. *plantarum* ATCC 14917. This investigation provides a better understanding of *L*. *plantarum*’s adhesion mechanisms under initial pH stress.

## Introduction

Lactic acid bacteria (LAB) fermentation is the simplest and safest way of preserving food. As traditional strains in food fermentation, especially in dairy, fermented meat and vegetable products [1], LAB can produce lactic acid to extend the shelf life of food and provide beneficial effects to human beings by improving the body’s natural defense system and regulating the gastrointestinal tract’s (GIT) micro-ecological balance. As important members of LAB, *L*. *plantarum* are always found in fermented plant-based foods and have many important physiological functions. *L*. *plantarum* 299v decreases fibrinogen concentrations in blood and increases carboxylic acids in feces [2]. *L*. *plantarum* WLPL04 exhibits strong antimicrobial activity against various pathogens [3]. The metabolites of *L*. *plantarum* R315 exert antioxidant and antibacterial activities [4], while *Lactobacillus plantarum* EM, isolated from kimchi, has shown high cholesterol removal by growing, resting, and even dead cells [5].

Bacterial adherence to intestinal epithelial cells or mucus is frequently considered a feature for a probiotic strain, since it can promote GIT residence time and interaction with hosts resulting in probiotic effects [6–8]. *L*. *plantarum*, a widely distributed LAB, is found in a range of environmental niches [9], including yogurt [10], cheese [11, 12], sausage [13, 14], olives [15], cocoa beans [16] and wine [17]. The usual initial environment of *L*. *plantarum* is a weak acid or weak alkaline in fermented food [18–20]. The pH value of the small and large intestines are 4.92 to -7.52 and 5.6 to -8.66, respectively [21]. Therefore, it is important to study the effects of initial acid and alkali stress on the adhesion activity of *L*. *plantarum*.

Metabolomics is the study of the global metabolite profiles of a cell under a given set of conditions [22]. As metabolic products reflect the interaction between the bacteria and its environment, metabolomics provides an unbiased assessment of a cellular state in the context of the particular condition [23, 24]. Ultra-Performance Liquid Chromatography (UPLS)-Electrospray ionization (ESI)-Quadrupole-Time of Flight (Q-TOF)-mass spectrometry (MS) is one of the most effective analytical tools for the study of metabolomics, and research has shown that HILIC-ESI-Q-TOF MS can provide the maximum amount of information about center carbon cycle metabolism [25]. For metabolomics, principal component analysis (PCA), orthogonal projection to latent structure-discriminant analysis (OPLS-DA) and hierarchical clustering are three effective analysis approaches. PCA and hierarchical clustering are untargeted approaches. PCA reflects the variation between and within the sample groups in general. Hierarchical clustering visually displays the samples relationship and expression pattern differences of metabolites in different samples. OPLS-DA, a targeted approach, establishes the relationship model between the metabolite expression and the sample category.

Although it has long been recognized that acid stress affects adhesion ability, and many researchers have studied LAB’s acid-adaption mechanism [26–30], the metabolic details related to initial pH stress and its effect on the adherence activity of LAB remain largely undefined. The present study seeks to identify the relationship between the adhesion activity of, and metabolic changes in, *L*. *plantarum* ATCC 14917 under initial acid and alkali stress.

## Materials and methods

### pH changes and growth curves at different initial pH levels

*L*. *plantarum* ATCC 14917 was purchased from the China General Microbiological Culture Collection Center and grown at 37°C in MRS broth with different initial pH levels (5.5, control (6.2), 8.5). Then the optical density and the pH were measured every 3 hours using a microplate reader at 600 nm (Tecan infinite M200 PRO, Switzerland) and a pH indicator (FE20-FiveEasy Plus™).

### Scanning electron microscope (SEM) and transmission electron microscope (TEM) analysis

After *L*. *plantarum* ATCC 14917 were cultured at different initial pH levels for 20 h at 37°C, the bacteria were harvested by centrifugation (5000 g, 5 min, 4°C). For SEM, 2.5% glutaraldehyde was used to fix the bacteria for 2 h and washed three times with phosphate buffer solution (PBS, pH 7.2). Then, the samples were dehydrated for 15 min in ethanol at concentrations of 30%, 50%, 70%, 80%, 90% and 100%, and replaced for 10 min with tertiary-butanol alcohol solution (25%, 50%, 75%, 100% v/v). Each process of dehydration and replacement involved centrifugation (7000 g, 5 min, 4°C). Samples were freeze-dried for 24 h (Christ, Alpha 1–4 LD plus, Germany). Finally, the samples were covered with a layer of gold palladium^9^ (Carbon Coating Unit, E-1010/E-0635, Hitachi, Japan) and viewed with a Hitachi S-3400N (Japan) at 10.0 kV in high-vacuum mode. The samples for TEM were carried out according to the method described by Deyi Xu [31] with some modifications. The samples were fixed in phosphate-buffered 4% glutaraldehyde for 12 hours (4°C), washed three times in phosphate buffer (5000 g, 15 min, 4°C), and fixed in 1% osmic acid for 2 h. Then they were quickly washed three times in phosphate buffer (4°C), dehydrated 20 min in a graded alcohol series (30%, 50%, 70% and 90%) and further dehydrated 20 min in graded acetone (90% and 100%). Then the samples were washed three times in absolute acetone, infiltrated (1 h) by low viscosity embedding media 1:1 and 2:1, and left overnight before they were embedded in pure Spurr's mixture and polymerised at 37°C for 12 h and at 60°C for 72 h. Ultrathin sections were cut and stained. Then the bacteria were observed with a Hitachi model H-800 transmission electron microscope, using an accelerating voltage of 200 kV.

### Aggregation and adhesion ability

*L*. *plantarum* ATCC 14917 was grown until the late exponential phase (20 h) in MRS broth at different initial pH levels (5.5, control (6.2), 8.5). After 20 h growth at 37°C, the bacteria were harvested by centrifugation (5000 g, 5 min, 4°C) and washed twice with phosphate buffer solution (PBS, pH 7.2). The supernatant was removed by centrifugation (5000 g, 5 min, 4°C) and the precipitate of *L*. *plantarum* ATCC 14917 were preserved at -80°C for further use.

*L*. *plantarum* ATCC 14917 were suspended in PBS to 0.5 optical density value at 600 nm [32]. The bacteria suspension (2 ml) was placed in centrifuge tubes. After incubation at 37°C for 2 h, 1 ml of the upper suspension was transferred to other centrifuge tubes and the OD values were measured using a microplate reader at 600 nm (M200 PRO, TECAN, Switzerland). Aggregation was calculated using the following formula:

$$Aggregation\;(\%)=(1-\frac{{OD}_{upper}}{{OD}_{total}})\times 100\%$$

Adhesion activity of *L*. *plantarum* to mucin was carried out according to the method of Antikainen and Uroić with some modifications [33, 34]. The type II mucin (from porcine stomach, Sigma-Aldrich, USA) was diluted to a concentration of 2 mg/mL with PBS (pH 7.2, 50 mM). One hundred μL of the type II mucin was added to each well of 96-well flat bottom plates and incubated at 37°C for 1 h. Then the mucin was washed three times with PBS to remove any unbound mucin and the plates were dried (55°C, 20 min). One hundred μL of *L*. *plantarum* suspension (OD_600_ of 1.0) was added to each well and incubated at 37°C for 3 h. Then any non-adhered bacteria were removed three times with 200 μL of PBS plus 0.05% Tween 20 (PBST). The adhered bacteria were detected by crystal violet staining (1 mg/ml, 45 min). The plates were washed three times with PBS to remove any excess crystal violet. Then PBS (100 μL/well) was added to the 96-well plates and incubated at 37°C for 1 h before OD value measurement (595 nm). Adhesion activity is directly proportional to OD value.

For adhesion activity to HT-29 cell lines from human colon adenocarcinoma [35], the *L*. *plantarum* bacteria (OD_600_ of 1.0) were resuspended in carbonate buffer solution at 37°C for 2 h in the dark for FITC labeling. The bacteria were harvested by centrifugation (5000 g, 5 min, 4°C) and washed with PBS (pH 7.2). Then 1% paraformaldehyde was used to fix the bacteria for 30 min, which were then washed twice with PBS (pH 7.2). The bacteria were resuspended in fresh McCoy's 5A medium and added to the prepared HT-29 monolayers to be cultured in 6-well plates at 37°C with 5% CO_2_ for 2 h. The monolayers were then washed 5 times with PBS to remove any unbound bacteria. A fluorescent microscope was used to observe the bacteria adhering to the HT-29 monolayers. Each assay was conducted at the same time in three independent experiments.

### Gene expression of adhesion-related proteins

Total RNA was extracted from the *L*. *plantarum* (late exponential phase, 20 h) using a Bacterial RNA Kit (OMEGA Biotek, USA). The reverse transcription of the RNA was performed with a TransScript All-in-One First-Strand cDNA Synthesis Super Mix kit (One-Step gDNA Removal, TransGen Biotech, China). The qPCR was implemented in a TransStart Tip Green qPCR SuperMix kit (TransGen Biotech, China) and quantified with a LightCycler96 (Roche, Switzerland). The quantitative PCR cycle threshold (CT) results were analyzed using the comparative CT method (2^-ΔΔCT^ method) with some modifications. All kits were used according to the manufacturers’ instructions. The genes of adhesion-related proteins of *L*. *plantarum* ATCC 14917 contained mannose-specific adhesion (*msa*), mucus-binding protein (*mub*1, *mub*2, *mub*3 and *mub*4), lipoprotein signal peptidase (*lsp*A) and elongation factor thermo unstable EF-Tu (*tuf*). S1 Table lists the primers used in this study.

### Metabolism analysis

The harvested bacteria (late exponential phase, 20 h) were centrifuged (4000 g, 5 min) and washed twice with PBS (pH 7.2). After the supernatant was removed by centrifugation (4000g, 5 min, 4°C), the precipitate was dispersed in 1 ml precooling methanol/acetonitrile/water solution (2:2:1, v/v), and then vortex and ultrasonic broken at -20°C for 1 h into precipitated protein. The precipitate was then removed (12000 g, 15 min, 4°C) and the metabolites were lyophilized by vacuum-freeze drying. For mass spectrometry analysis, the lyophilized powder was dissolved with 100 μL acetonitrile solution (acetonitrile: water = 1:1, v/v) and centrifuged at 12000 g (4°C) for 15 min. The supernatant was analyzed using an UPLC (1290 Infinity LC, Agilent Technologies) equipped with a ACQUITY UPLC BEH Amide column (2.1×100 mm, 1.7 μm) coupled with a quadrupole time-of-flight mass spectrometer (AB Sciex TripleTOF 6600) set in positive-ionization mode.

The column temperature was set at 25°C with a flow rate of 0.3mL/min. Mobile phase A was composed of 25 mM ammonium acetate and 25 mM ammonia. Acetonitrile was used for mobile phase B. The gradient-elution procedure was as follows: 0–1 min, 85% mobile phase B; 1–12 min, B linear change from 85% to 65%; 12–12.1 min, B linear change from 65% to 40%; 12.1–15 min, B maintained at 40%; 15–15.1 min, B linear change from 40% to 85%; and 15.1–20 min, mobile phase B maintained at 85%. The samples were stored at 4°C and an automatic sampler was used during the analysis. ESI source conditions were as follows: ion source gas1 (Gas1): 60; ion source gas2 (Gas2): 60; curtain gas (CUR): 30; source temperature: 600°C; IonSapary Voltage Floating (ISVF)±5500 V; TOF MS scan range m/z: 60–1000 Da; product ion scan range m/z: 25–1000 Da; TOF-MS scan accumulation time: 0.20 s/spectra; product ion scan accumulation time: 0.05 s/spectra; secondary mass spectrometry was acquired by information-dependent acquisition (IDA) with a high-sensitivity model; declustering potential (DP):±60 V; collision energy: 35 + 15 for eV; IDA: exclude isotopes within 4 Da; and candidate ions monitored per cycle: 6.

The metabolism experiments were repeated five times and the quality control samples (QC samples) were analyzed in continuous random order. The raw data of metabolomics were converted to common data format (.mzML) files by Proteo Wizard. The program XCMS was used for peak alignment, retention time correction and extracting peak area. The data were extracted by XCMS and delete the ion peak (missing value> 50%) in the group. Then SIMCA-P 14.1 (Umetrics) was used for multivariate statistical calculations and plotting.

### Fatty acids analysis

The membrane fatty acid composition of the *L*. *plantarum* ATCC 14917 was determined using gas chromatography according to the method of Garbay and Delettrea [36, 37] with some modifications. Fatty acid methyl esters were extracted with 2 mL of hexane and shaken for 20 s. After decanting for 5 min, the upper phases were stored at -80°C in an airtight glass bottle for further use. Analyses were performed on a gas chromatographer (Aglient 7890B-5977A, USA). A capillary column (DB-WAX, 30 m × 250 μm × 0.25 μm, Agilent 122–7032, USA) was used. The fatty acid methyl esters were identified by using the NIST library and by comparing their retention times with Bacterial Acid Methyl Esters Mix (Sigma, 47080-U). The ratio between unsaturated and saturated fatty acids (U/S) was determined.

### Statistical analysis

The data of auto-aggregation, adhesion to mucin and HT-29 cell lines and the membrane fatty acids were analyzed using the SAS 8.0 program and expressed as means±stand deviation (SD). The statistical differences in the groups were analyzed by one-way analysis of variance with a significance of P <0.05. All curves were fitted using Origin 9.0 software.

The data of metabolomics were analyzed by multivariate statistical analysis and one-way analysis of variance. PCA and OPLS-DA combined with T-test were used to screen the significant difference metabolites. Metabolite identification used precise mass matching (<25 parts per million (PPM)), secondary spectrogram matching, and an in-house retrieval database. The obtained difference metabolites were submitted to KEGG website for the analysis of related metabolic pathways.

## Results and discussion

### Changes in pH and growth of *L*. *plantarum* ATCC14917

**Fig 1:**
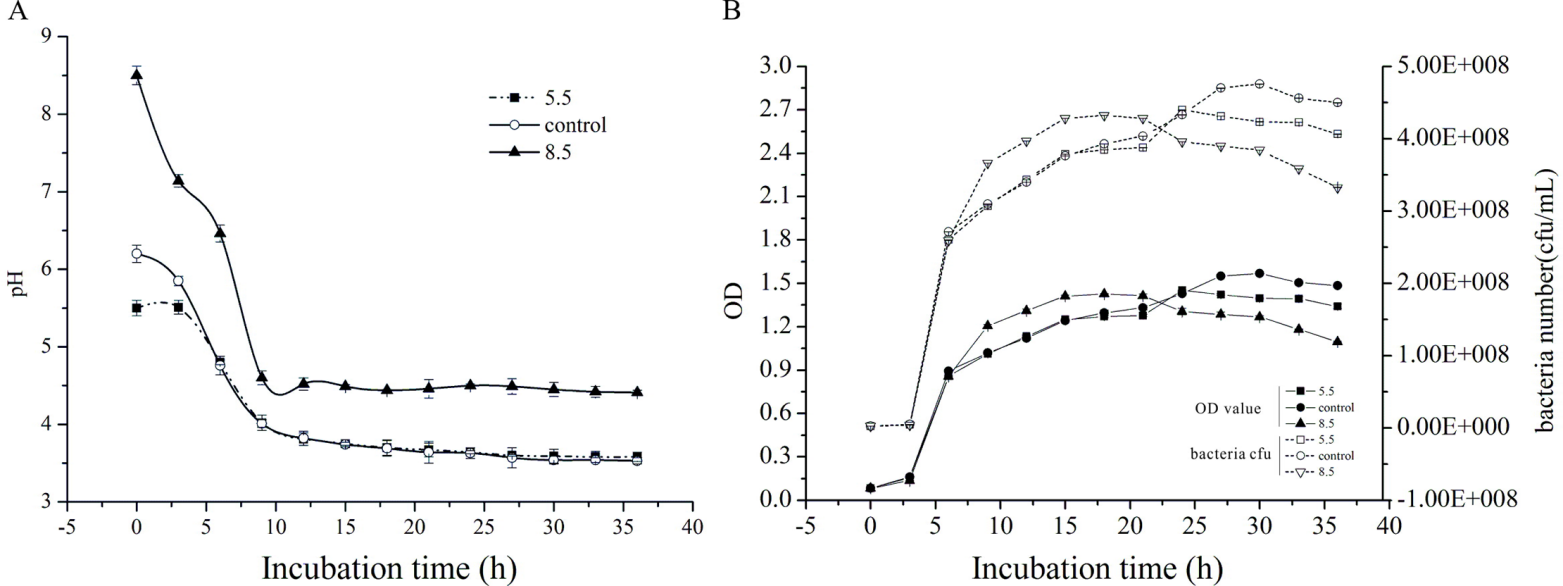
pH changes (A) and growth (B) of *L*. *plantarum* ATCC 14917 in acid, control and alkali groups.

Fig 1 shows changes in pH and growth of *L*. *plantarum* ATCC 14917 from the control (pH 6.2), acid (pH 5.5) and alkali (pH 8.5) treated samples. The pH value of the three groups decreased dramatically when incubated for 5 hours (Fig 1A). The acid (pH 5.5) and control groups (pH 6.2) had a lower pH value (3.5) when bacteria entered the stationary phase (after 12 h). The number of bacteria in the alkaline group was a little more than in the acid and control groups during the middle and later exponential phase (Fig 1B). The number of bacteria in the alkaline group decreased during the stationary phase, but was stable in the control and acid groups. These results show that the decline phase of *L*. *plantarum* ATCC14917 occurs early under initial alkali stress.

### SEM and TEM analysis of *L*. *plantarum* ATCC14917

**Fig 2:**
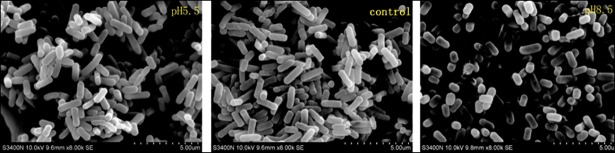
SEM images of *L*. *plantarum* ATCC 14917 in acid, control and alkali groups.

**Fig 3:**
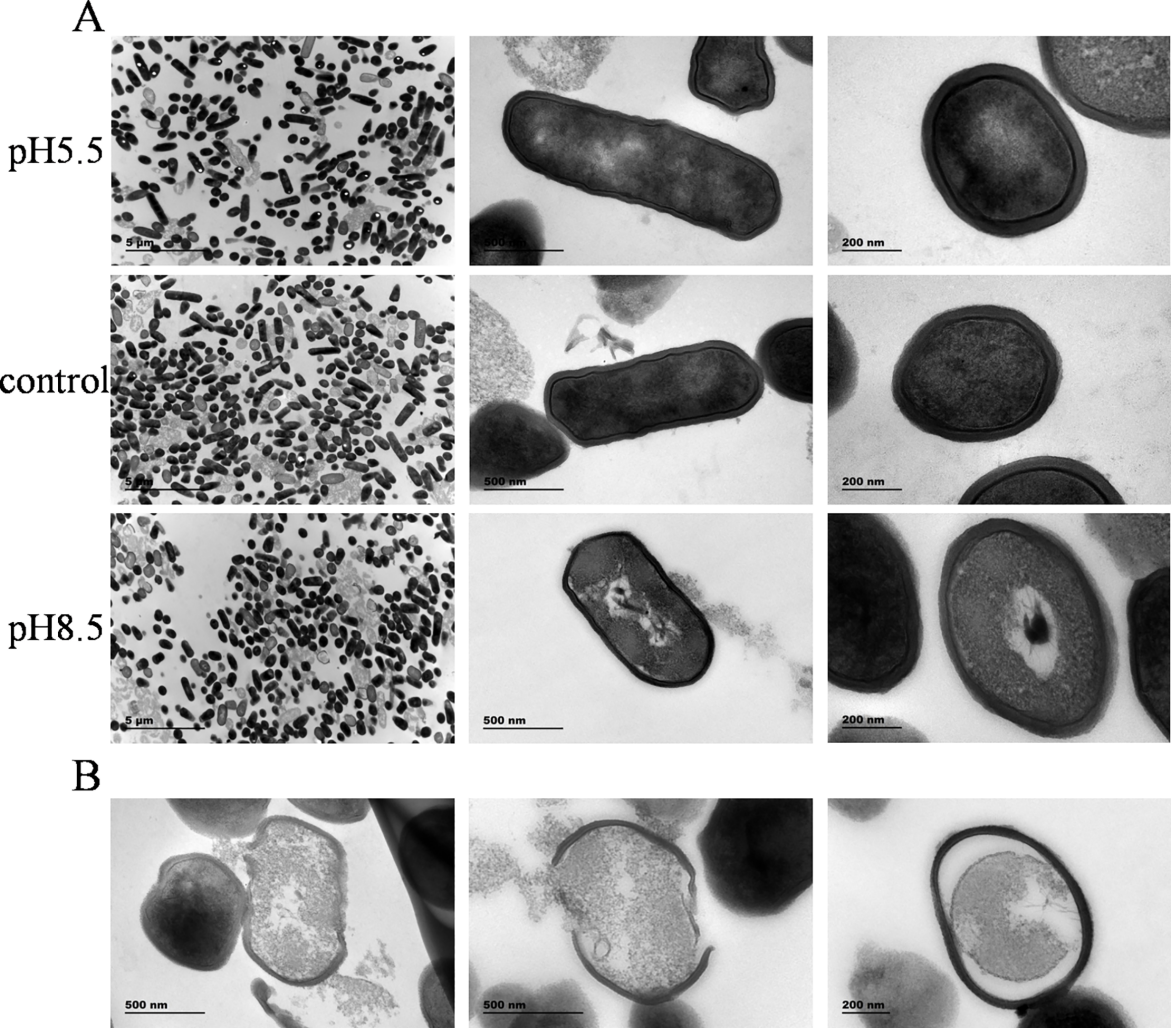
TEM images of *L*. *plantarum* ATCC 14917 in acid, control and alkali groups.

With the help of SEM and TEM, the morphological changes of *L*. *plantarum* ATCC14917 cells were clearly observed. The pictures showed that the mycelia morphology of *L*. *plantarum* ATCC14917 cells was long rods with slender thalli in the acid and control groups (Fig 2A and 2B), but was short rods with thickset thalli in the alkali group (Fig 2C). Under initial alkali stress, some *L*. *plantarum* ATCC14917 were broken, autocytolytic, cell-wall deficient, and the cell walls were thin (Fig 3). Due to the initial alkali stress, the *L*. *plantarum* ATCC14917 was not suited to the acidic environment after the stationary phase.

### *L*. *plantarum* ATCC 14917 aggregation and adhesion ability

**Fig 4:**
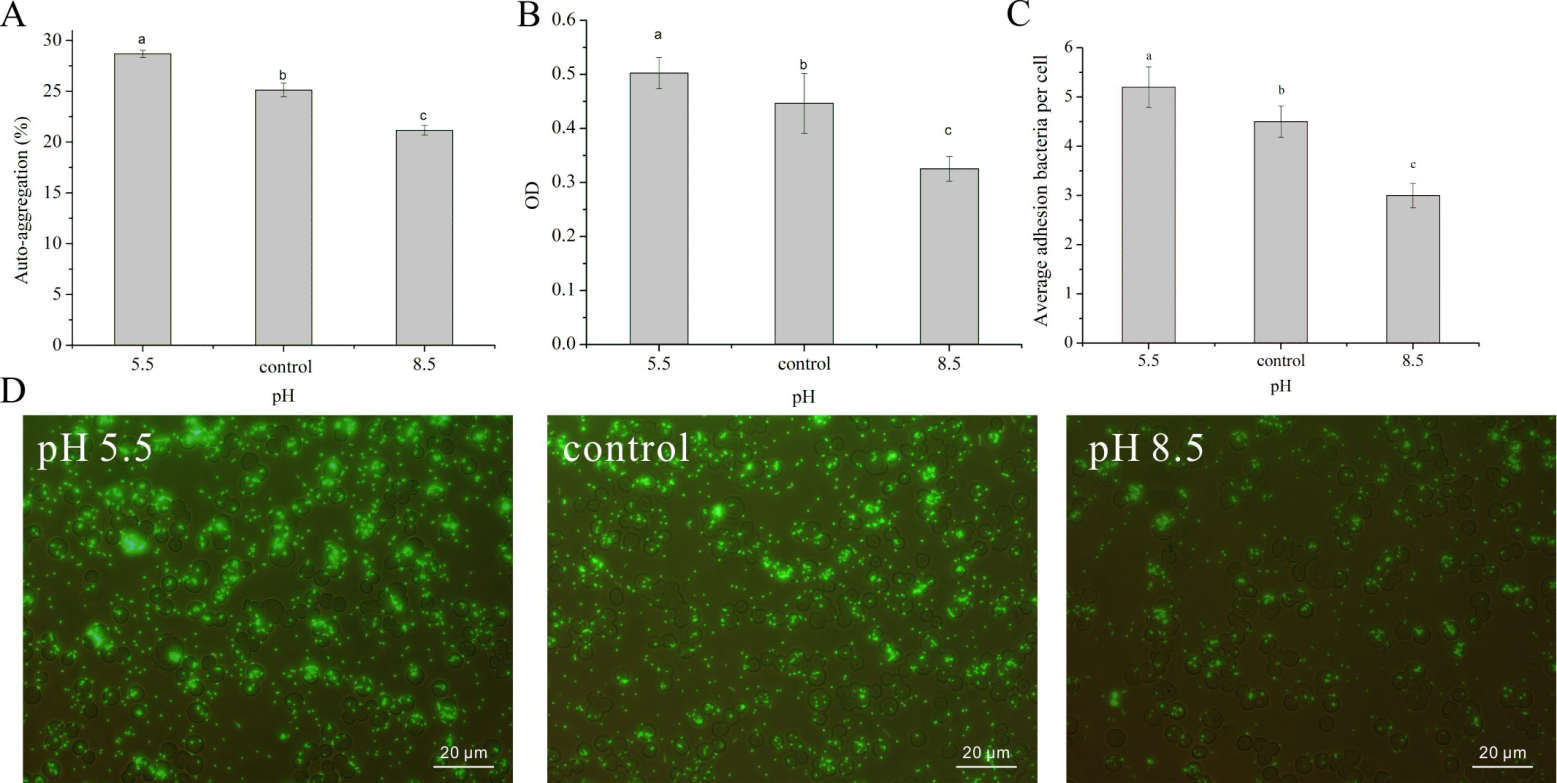
Aggregation and adhesion ability of *L*. *plantarum* ATCC 14917 under initial pH stress. A, auto-aggregation of *L*. *plantarum* ATCC 14917; B, adhesion to type II mucin; C and D, adhesion to HT-29 cells.

As shown in Fig 4, the auto-aggregation and the adhesion ability of *L*. *plantarum* ATCC 14917 to mucin and HT-29 cells was lower in the alkali group than in the acid and control groups (Fig 3A, 3B and 3C). The average number of *L*. *plantarum* ATCC 14917 adhering to HT-29 cells was 5.2 (acid group), 4.5 (control group) and 3.0 per cell (alkali group). These results agree with the findings of another study, which found that high auto-aggregation ability is related to strong adhesion ability [38].

### Gene expression of adhesion-related proteins

**Fig 5:**
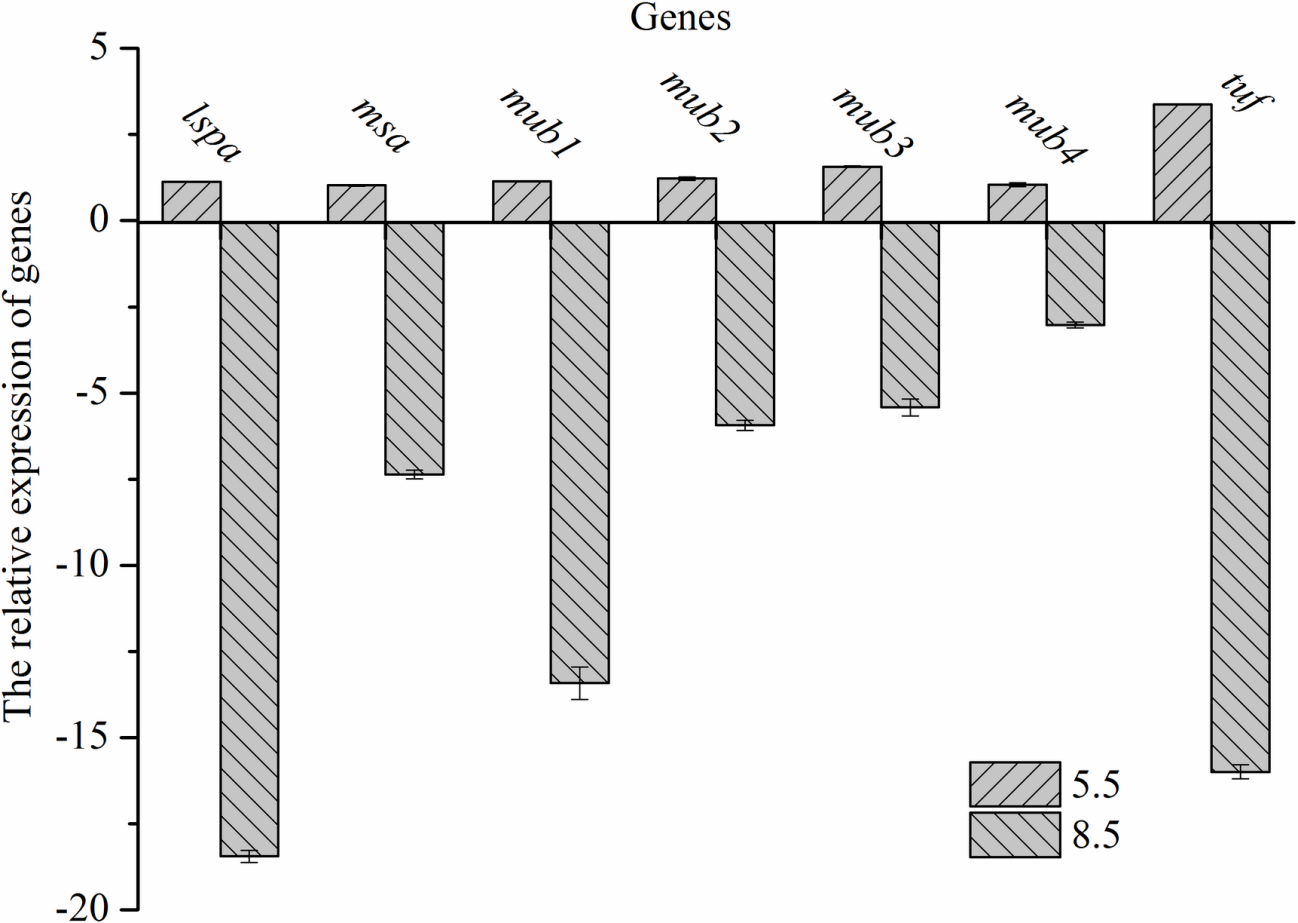
mRNA expression levels of *L*. *plantarum* ATCC 14917 adhesion-related proteins under initial pH stress.

To further confirm the adhesion ability of *L*. *plantarum* ATCC 14917 under initial acid and alkali stress, RT-qPCR analysis was conducted. As shown in Fig 5, the results of RT-qPCR showed that the gene expression of adhesion-related proteins was up-regulated in the acid group but down-regulated in the alkali group. The relative expression of *msa*, *mub*1, *mub*2, *mub*3, *mub*4, *lsp*A and *tuf* of *L*. *plantarum* ATCC 14917 under initial acid stress were 1.13, 1.03, 1.14, 1.22, 1.57, 1.04 and 3.38. The relative expression of *msa*, *mub*1, *mub*2, *mub*3, *mub*4, *lsp*A and *tuf* under initial alkali stress were -18.45, -7.36, -13.42, -5.94, -5.42, -3.02 and -15.99.

### Metabolism analysis

#### Statistical analysis of the UHPLC-Q-TOF MS metabolic data

Using UHPLC-Q-TOF MS analysis, the TIC chromatograms of the intracellular metabolites of *L*. *plantarum* ATCC 14917 from the QC samples (S1 Fig), and the acid, control and alkali groups (S2 Fig) were obtained. The response intensity and retention time of the chromatographic peaks from four analyses almost overlap, which showed that the variation caused by any instrument error was small. The TIC chromatogram from acid, alkali and control groups showed a strong signal, high peak capacity and good retention time.

**Fig 6:**
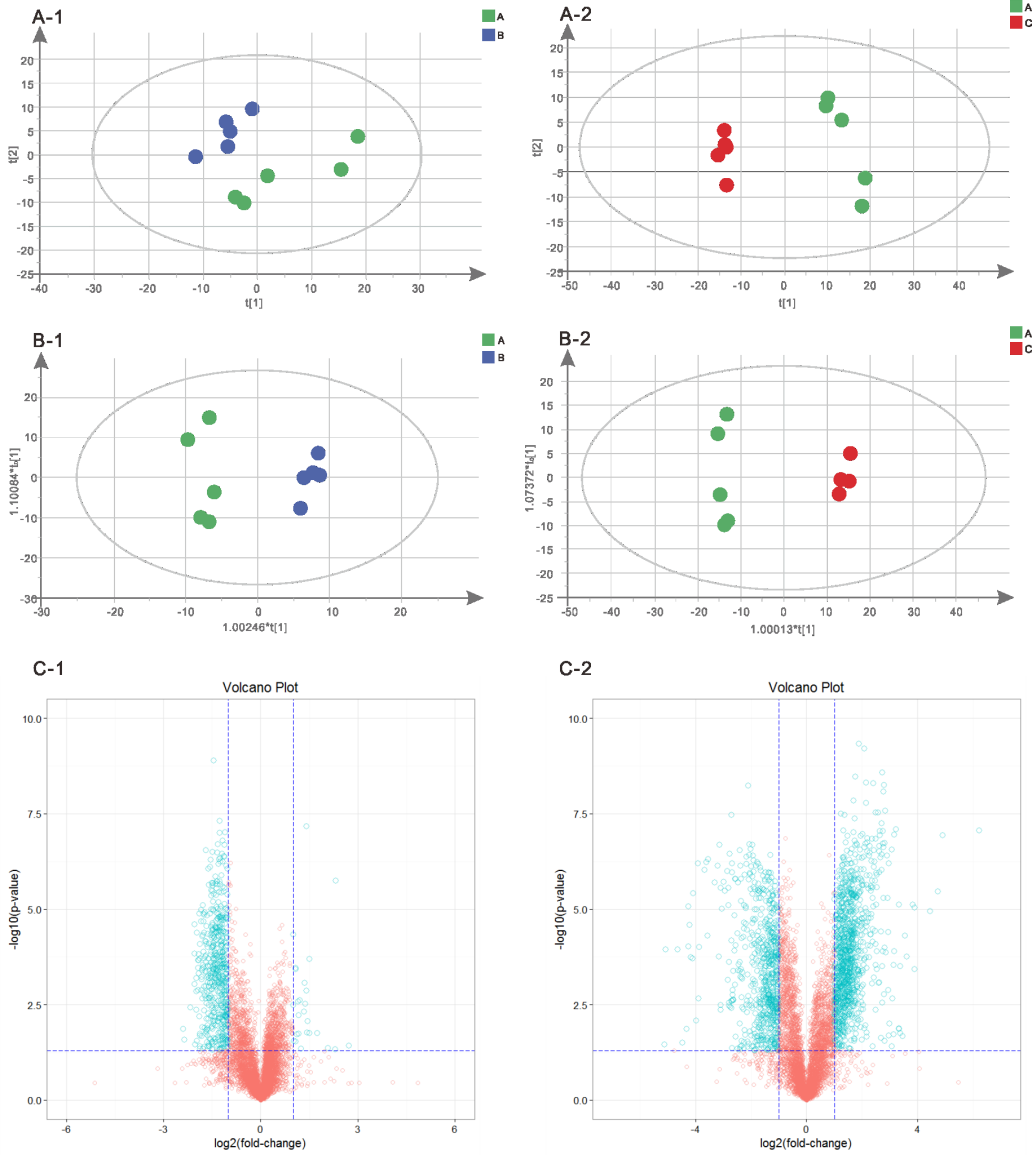
Metabolism analysis of *L*. *plantarum* ATCC 14917 under initial pH stress. A-1, PCA score plot of acid and control groups; A-2, PCA score plot of alkali and control groups; B-1, OPLS-DA score plot of acid and control groups; B-2, OPLS-DA score plot of alkali and control groups; C-1, volcano plot of acid and control groups; C-2, volcano plot of alkali and control groups.

A clearer insight into the effect of initial acid and alkali stress on the metabolite profile of *L*. *plantarum* ATCC 14917 was gained by conducting multivariate statistical analysis. The PCA scores plot (Fig 6A) showed clear variation in the metabolite profiles under initial acid and alkali stress. We can reliably interpret the metabolic difference of samples based on the illustration from the PCA. Simultaneously, OPLS-DA showed good model quality and obvious intergroup metabonomic differences (Fig 6B). The values for R2X and Q2 (S3 Table) indicated that the three models were of reasonable quality, can eliminate and classify unrelated noise information, and can obtain more reliable information about the metabolites which displayed significant differences. Furthermore, the blue point (Fig 6C) from the single variable statistical analysis of a Volcano Plot Analysis (combine a Fold Change Analysis (FC) and a T test) indicated significantly different metabolites (FC > 2.0 and P < 0.05).

The Variable Importance for the Projection (VIP, VIP obtained by OPLS-DA model) value combined with P Value were used as a screening criteria to got the significant difference metabolites. If a metabolite meets the condition of VIP>1 and P value < 0.05, it is a significant differential metabolite. If a metabolite meets the condition of VIP>1 and 0.05<P value<0.1, it is a differential metabolite. All these differential metabolites were given in S1 Table. Compared with the control group, the intracellular metabolism of *L*. *plantarum* ATCC 14917 was significantly influenced by the initial pH stress. Under the initial acid stress, the differential metabolites of *L*. *plantarum* ATCC 14917 were dominated by 16 metabolites, including 1 fatty acid (trans-vaccenic acid), 1 amino acid (L-Histidine) and 14 metabolites involved in carbohydrate metabolism. Under the initial alkali stress, the differential metabolites were dominated by 38 metabolites, including 1 fatty acid (trans-Vaccenic acid), 12 amino acids and 25 metabolites involved in carbohydrate metabolism. Twelve kinds of up-regulated metabolites and 4 kinds of down-regulated metabolites were found in the acid group. Eleven kinds of up-regulated metabolites and 27 kinds of down-regulated metabolites (10 amino acids and 17 involved in energy metabolism) were found in the alkali group.

#### Cluster and metabolic pathway analysis

**Fig 7:**
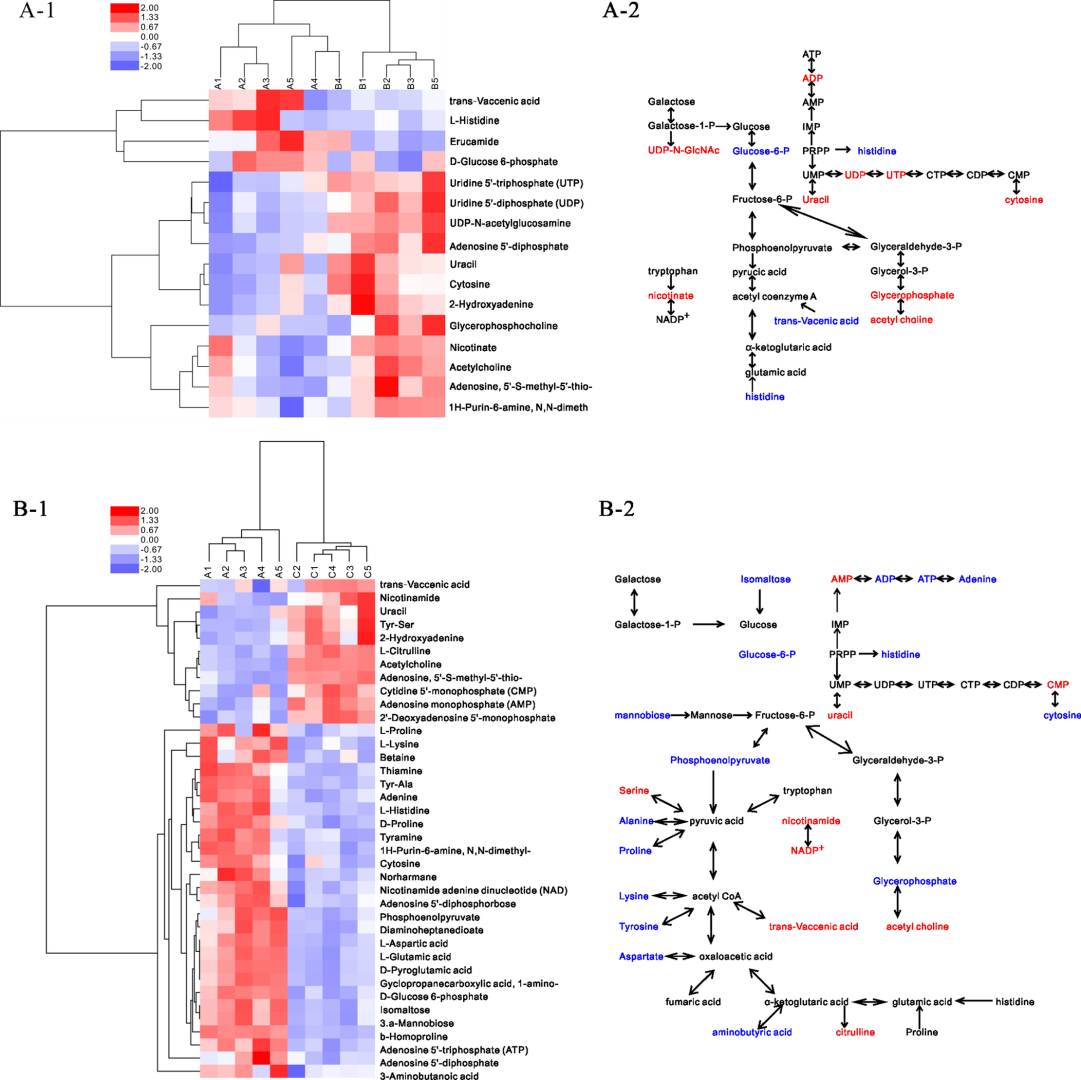
Clustering and metabolic pathways related to the different metabolites of *L*. *plantarum*. A, heatmap and metabolic pathway of acid and control groups; B, heatmap and metabolic pathways of alkali and control groups.

Hierarchical clustering (Fig 7A-1 and 7B-1) was conducted to assist in accurately screening for the marker metabolites. The related metabolic processes were then studied (Fig 7A-2 and 7B-2). Most of the differential metabolites were mainly involved in carbohydrate metabolism and were up-regulated in the acid group. In the alkali group, the differential metabolites were mainly involved in carbohydrate and amino-acid metabolism and were down-regulated.

The growth process of LAB generates acidic products that accumulate in the extracellular environment [39]. Therefore, the greater part of their growth metabolism occurs under acidic conditions. To adapt to acid stress, LAB have special pH-homeostasis mechanisms [40, 41], such as the arginine deiminase (ADI) pathway, glutamate decarboxylases (GAD) pathway and F0F1-ATPase proton pump.

In the ADI pathway, arginine is converted into citrulline and ornithine, while NH_3_ is generated, which reacts with H^+^ to alkalize the environment. In the alkali group, the amount of citrulline (fold changes 5.579) increased significantly, which indicated that the ADI pathway was suppressed. The GAD pathway involves lysine, arginine and glutamate, which transform into cadaverine, agmatine and aminobutyrate to control the pH of the bacterial environment by consuming hydrogen ions [42–44]. The amount of lysine (fold changes 0.565), glutamic acid (fold changes 0.530) and aminobutyrate (0.823) decreased in the alkali group, which mean that the GAD pathway was weakened by initial alkali stress. However, the amino acids involved in the ADI and GAD pathways did not appear to change in the acid group.

A previous study [45] showed that F0F1-ATPase facilitates the extrusion of protons from the cell cytoplasm to maintain pH homeostasis, but that energy is needed for this process to occur. Differing from the weakened energy metabolism found in the alkali group, the *L*. *plantarum* ATCC 14917 in the acid group displayed vigorous energy metabolism during the exponential phase (20 h). Therefore, the *L*. *plantarum* ATCC 14917 in the acid group displayed stronger pH homeostasis ability and were better suited to the acidic environment. However, the F0F1-ATPase activity had one drawback; the consumption of energy will prevent bacteria growth [46]. Our study supported this finding; the total number of *L*. *plantarum* ATCC 14917 in the acid and control groups were less than that found in the alkali group during the middle and later exponential phases (Fig 1B). It is assumed that vigorous energy metabolism and stronger pH-homeostasis ability promote the adhesion of *L*. *plantarum* ATCC 14917 *in vitro*.

### Cell-membrane fatty acid composition analysis

**Fig 8:**
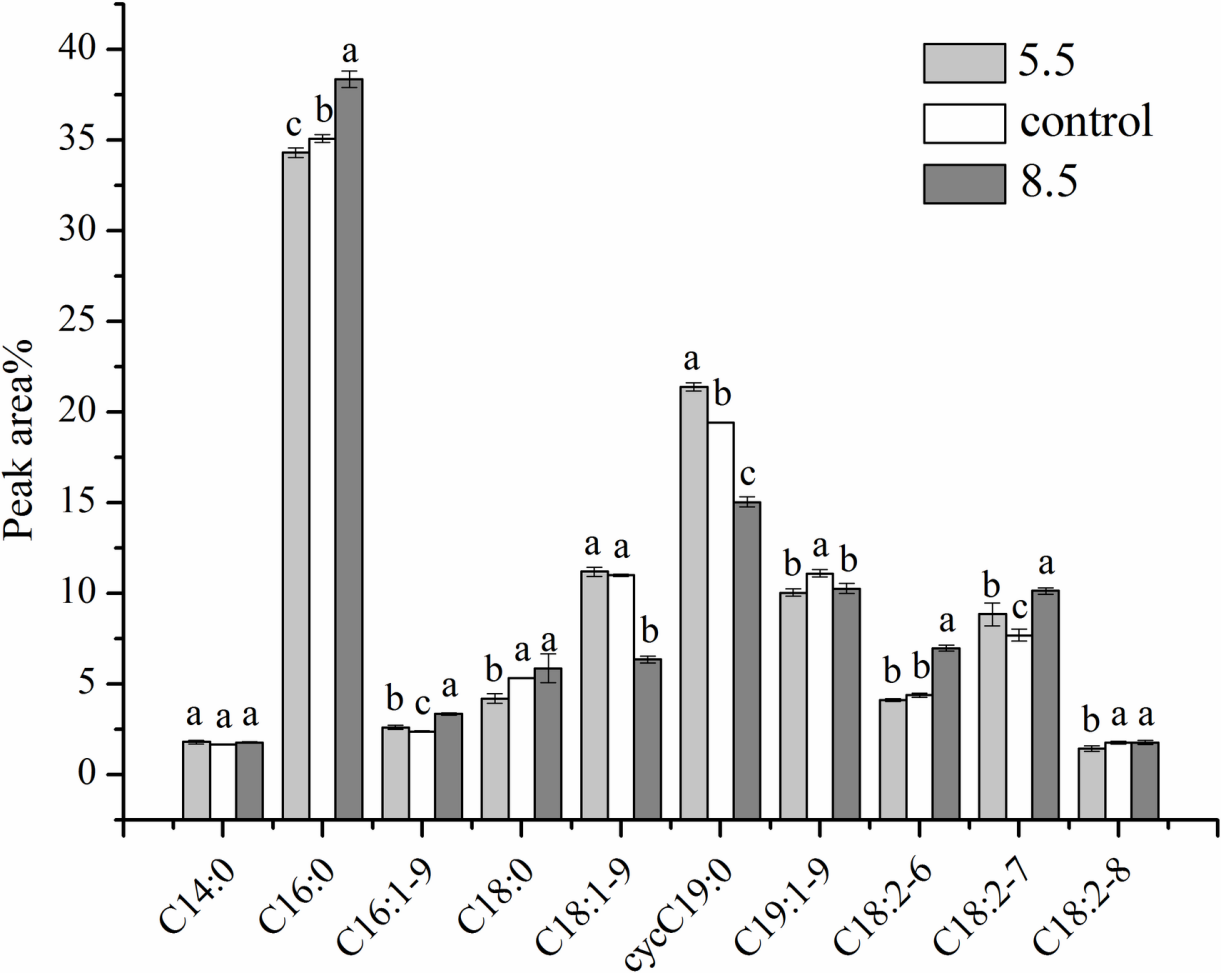
Cell membrane fatty acid composition analysis.

GC was performed to investigate the change in the cell membrane fatty acid composition of *L*. *plantarum* ATCC 14917 under initial acid and alkali stress (Fig 8). Ten fatty acids made up the membrane of *L*. *plantarum* ATCC 14917. The peaks were identified as tetradecanoic (myristic)(C14:0), hexadecanoic (palmitic)(C16:0), hexadecenoic (palmitoleic)(C16:1), octadecanoic (stearic)(C18:0), cis-9-octadecenoic (C18:1–9), octadecadienoic (C18:2–6, C18:2–7and C18:2–8), cis-9-neoctadecenoic (C19:1–9), and methyleneoctadecenoic (dihydrosterculic) (ΔC19:0–9) acid. Cyclopropane fatty acids accounted for 21.39% (acid group), 19.42% (control group) and 15.06% (alkali group) of the total cell membrane fatty acids. The cyclopropane fatty acid ΔC19:0–9 is produced by methylation of oleic acid and is considered unsaturated. Unsaturated fatty acids accounted for 59.67% (acid group), 57.91% (control group) and 53.97% (alkali group) of the total cell membrane fatty acids.

Unsaturated and cyclopropane fatty acids promote exchanges between extracellular and intracellular media by enhancing membrane permeability [47]. Dihydrosterculic acid (ΔC19:0–9) is responsible for the elasticity and flexibility of cell membranes. Furthermore, the high unsaturated/saturated fatty acids (U/S) ratio of LAB cell membranes can improve the liquidity of cell membranes and promote cell extracellular H^+^ eduction to maintain intracellular pH balance [48]. Sarkis[49] indicated that the binding characteristics of colon cancer cells may be determined in part by the membrane’s fatty acid composition. The high percentage of unsaturated and cyclopropane fatty acids in the acid and control groups resulted in better cell-membrane fluidity and acid-resistance ability. These properties contributed to the adhesion ability of *L*. *plantarum* ATCC 14917.

## Conclusion

This investigation illustrated the relationship between the adhesion ability of, and metabolism changes in, *L*. *plantarum* ATCC 14917 under initial acid and alkali stress. During initial acid stress, the energy metabolism of *L*. *plantarum* ATCC 14917 was vigorous with a higher unsaturated fatty acid content in the cell membranes. The acid resistance ability was stronger and adhesion ability was enhanced compared with the strains in the initial alkali stress conditions. Furthermore, the initial alkali stress did some damage to the cell membranes and weakened the energy metabolism, which decreased the adhesive ability of the *L*. *plantarum* ATCC 14917. Our study revealed that pH-mediated adhesion activity in *L*. *plantarum* ATCC 14917 is related to the metabolism of the related amino acids involved in an energy expenditure model.

## Supporting information

S1 Table Results of differential metabolites of acid stress vs. control and alkali stress vs. control. (PDF)

S2 Table Primers of genes of adhesion-related proteins used in this study. (PDF)

S3 Table The parameters (R2X and Q2) for evaluating the OPLS-DA model in this study. (PDF)

S1 Fig HILIC positive ion mode of control, acid and alkali group samples TIC chromatogram. (PDF)

S2 Fig HILIC-positive-ion mode of QC samples superimposed on TIC chromatogram. (PDF)
