# Supplementary material for: Metabolomics analysis of Lactobacillus plantarum ATCC 14917 adhesion activity under initial acid and alkali stress
Source: PLoS One. 2018 May 24;13(5):e0196231. doi: 10.1371/journal.pone.0196231 (PMC5967736; doi:10.1371/journal.pone.0196231)
Supplement: S1 Table — (PDF) [file pone.0196231.s001.pdf]

Table S1 Result of differential metabolites of acid stress vs. control and alkali stress vs. control

	Different metabolites	Fold changes
Acid stress *v.s.* control	Uridine 5'-triphosphate (UTP)	1.516625224
	Uridine 5'-diphosphate (UDP)	1.628879823
	UDP-N-acetylglucosamine	1.696020766
	Cytosine	1.726965825
	Adenosine, 5'-S-methyl-5'-thio-	1.542024068
	Adenosine 5'-diphosphate	1.746858267
	2-Hydroxyadenine	2.122469339
	Uracil	3.641058471
	trans-Vaccenic acid	0.461504454
	Nicotinate	1.311625976
	L-Histidine	0.637528112
	Glycerophosphocholine	1.235119799
	Erucamide	0.295632625
	D-Glucose 6-phosphate	0.82561749
	Acetylcholine	1.908554266
	1H-Purin-6-amine, N,N-dimethyl-	1.548569608
Alkali stress *v.s.* control	Tyramine	0.340764726
	Tyr-Ala	0.067846219
	trans-Vaccenic acid	2.278905306
	Thiamine	0.477831275
	Phosphoenolpyruvate	0.504821931
	Norharmane	0.25150228
	Nicotinamide adenine dinucleotide (NAD	0.649373815
	L-Proline	0.655333803
	L-Lysine	0.565172049
	L-Histidine	0.57809759
	L-Glutamic acid	0.530338957
	L-Citrulline	5.579516857
	L-Aspartic acid	0.38995919
	Isomaltose	0.523501562
	D-Pyroglutamic acid	0.522787811
	D-Proline	0.456420159
	Diaminoheptanedioate	0.509784529
	D-Glucose 6-phosphate	0.382526981
	Cytosine	0.58654247
	Cytidine 5'-monophosphate (CMP)	1.676187521
	Cyclopropanecarboxylic acid, 1-amino-	0.545038058
	Betaine	0.812169043
	Adenosine, 5'-S-methyl-5'-thio-	2.435056997
	Adenosine monophosphate (AMP)	2.181652636
	Adenosine 5'-triphosphate (ATP)	0.401893068
	Adenosine 5'-diphosphoribose	0.69209147

Adenosine 5'-diphosphate	0.738017515
Adenine	0.309655484
Acetylcholine	74.25914333
3-Aminobutanoic acid	0.823231704
3.alpha.-Mannobiose	0.531887773
2-Hydroxyadenine	3.962232885
2'-Deoxyadenosine 5'-monophosphate	1.786893883
1H-Purin-6-amine, N,N-dimethyl-	0.224726062
.beta.-Homoproline	0.06640042
Uracil	8.523925987
Tyr-Ser	16.46449208
Nicotinamide	2.565405085
