# Supplementary material for: Metabolomics analysis of Lactobacillus plantarum ATCC 14917 adhesion activity under initial acid and alkali stress
Source: PLoS One. 2018 May 24;13(5):e0196231. doi: 10.1371/journal.pone.0196231 (PMC5967736; doi:10.1371/journal.pone.0196231)
Supplement: S2 Table — (PDF) [file pone.0196231.s002.pdf]

Table S2 Primers of genes of adhesion-related proteins used in this study

genes	primer (5'-3')
lsp F	GGGCTGCTTGGAGTATCTTA
lsp R	ATTCCCCAAAGTTCCCGCAA
msa F	GCAGCAGATTCCGAAGTG
msa R	CGCTGACCGCCTTATTGA
mub 1 F	CGGAAGTCCCTCTGAGCA
mub 1 R	AGTTGATGCGGAGGTTGTTT
mub 2 F	GATGTTATGGTTCCCACAGCAA
mub 2 R	TGTCGTCGCACCCATCGT
mub 3 F	AGATGCGGATGGAAAGCCACTT
mub 3 R	GCCCACTCGGCTCCGGCGTGT
mub 4 F	GTGACCAACGGCATGAGT
mub 4 R	AAGCATCTACCTGAGTCTC
tuf F	TGACTTAGGTGAAGCCGG
tuf R	GTGGACGATAGTTTGAG
