# Supplementary material for: Metabolomics analysis of Lactobacillus plantarum ATCC 14917 adhesion activity under initial acid and alkali stress
Source: PLoS One. 2018 May 24;13(5):e0196231. doi: 10.1371/journal.pone.0196231 (PMC5967736; doi:10.1371/journal.pone.0196231)
Supplement: S3 Table — (PDF) [file pone.0196231.s003.pdf]

Table S3 The parameters (R2X and Q2) for evaluating the OPLS-DA model in this study

Samples	Model	R2X(cum)	Q2(cum)
B-A	OPLS-DA	0.572	0.821
C-A	OPLS-DA	0.758	0.983

R2: represents the model interpretation rate;Q2: presentation model prediction ability
R2 and Q2 are more closer to 1, the model is the more stable and reliable
