# Supplementary figures and images for: Metabolomics analysis of Lactobacillus plantarum ATCC 14917 adhesion activity under initial acid and alkali stress
Source: PLoS One. 2018 May 24;13(5):e0196231. doi: 10.1371/journal.pone.0196231 (PMC5967736; doi:10.1371/journal.pone.0196231)

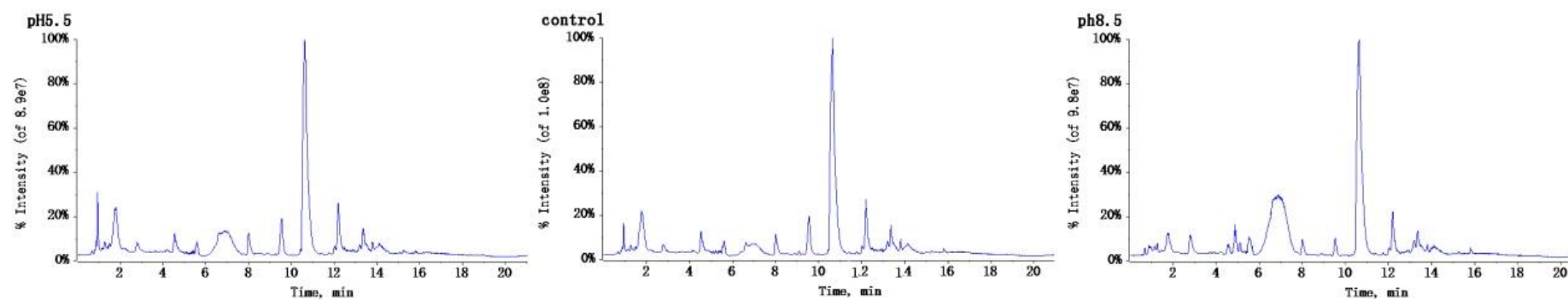

Figure S1. HILIC positive ion mode of control, acid and alkali group samples TIC chromatogram

Supplement: S1 Fig — (PDF) [file pone.0196231.s004.pdf]

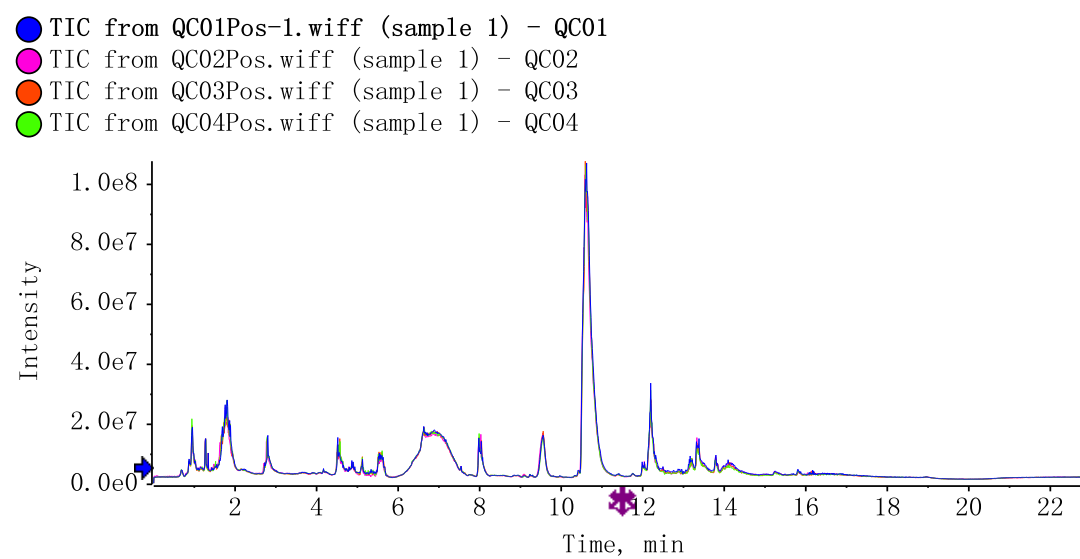

Figure S2. HILIC positive ion mode of QC samples superimposed TIC chromatogram

Supplement: S2 Fig — (PDF) [file pone.0196231.s005.pdf]
